# Benchtop High-MAS NMR for Paramagnetic Materials

**DOI:** 10.3390/molecules31122038

**Published:** 2026-06-11

**Authors:** Raiker Witter, Andres Oss, Radostina Stoyanova, Ago Samoson

**Affiliations:** 1 Laboratory of Spin Design, Department of Cybernetics, Tallinn University of Technology (TalTech), Ehitajate tee 5, 19086 Tallinn, Estonia; 2 Institute of Quantum Optics, University of Ulm (UUlm), Albert-Einstein-Allee 11, 89081 Ulm, Germany; 3 Institute of Nanotechnology, Karlsruhe Institute of Technology (KIT), P.O. Box 3640, 76021 Karlsruhe, Germany; 4 Helmholtz Institute Ulm (HIU), Helmholtzstraße 11, 89081 Ulm, Germany; 5 Institute of General and Inorganic Chemistry, Bulgarian Academy of Sciences, 1113 Sofia, Bulgaria

**Keywords:** magic-angle spinning (MAS), small-bore magnet, compact NMR instrumentation, high-speed spinning, low-field NMR, portable NMR systems, miniaturized MAS probe, permanent magnet

## Abstract

We report a compact benchtop solid-state NMR platform that achieves 50 kHz magic-angle spinning (MAS) in a 1.4 T permanent magnet with an 18 mm bore, enabling high-speed MAS under extremely space-constrained conditions. The probe architecture leverages field–bore orthogonality for convenient magic-angle alignment and is demonstrated with miniaturized 1.8 mm rotors (≈5 mm length) at stable high-speed operation. As a demanding test case, we measure ^7^Li MAS NMR of the paramagnetic layered cathode oxide LiNi_0.5_Mn_0.5_O_2_, where hyperfine interactions produce very large paramagnetic shifts spanning the several-thousand-ppm regime. Overall, the results establish a path toward portable, cost-effective high-MAS NMR in compact permanent-magnet geometries.

## 1. Introduction

Magic-angle spinning (MAS) solid-state NMR is a powerful tool for structural and dynamic analysis of solids ranging from inorganic functional materials to polymers and biomacromolecules [1]. Increasing the MAS frequency improves resolution by more efficiently averaging second-rank anisotropic interactions (e.g., dipolar couplings and chemical-shift anisotropy) and by reducing residual broadening from higher-order terms (e.g., second-order quadrupolar interactions), while also affecting relaxation [2,3].

At the same time, there is growing demand for portable, low-cost, and infrastructure-light NMR (e.g., for at-line process control, materials screening, education, and field deployable analysis), motivating renewed interest in permanent-magnet platforms. However, combining MAS with compact permanent magnets is not straightforward: permanent magnets typically provide lower *B*_0_, restricted bore sizes, and less flexible probe geometries than superconducting systems, all of which constrain rotor size, stable spinning, temperature control, and sample handling. Consequently, most compact-MAS demonstrations have historically been limited to modest spinning speeds and/or larger bores. Previous studies have reported MAS at modest speeds (up to ~10 kHz) in bore sizes larger than 25 mm using 1.0–1.5 T Halbach arrays or single-pole configurations [4,5].

In parallel, MAS at elevated speeds has emerged as a key enabling technology for modern solid-state NMR, providing substantial gains in resolution and sensitivity across a broad range of nuclei and materials classes. Comprehensive reviews have highlighted how fast/ultrafast MAS unlocks improved averaging and enables experiments that were previously impractical due to broad lines or short coherence lifetimes, especially when combined with modern pulse sequence design [6].

A particularly demanding and scientifically relevant class of targets for ultrafast MAS are paramagnetic energy materials, such as transition-metal (TM) oxide battery electrodes. In these systems, strong hyperfine interactions and fast relaxation can broaden resonances over hundreds to thousands of ppm, complicating both assignment and quantification. A foundational body of work has established that, despite these challenges, solid-state NMR provides uniquely local information on lithium environments, cation ordering, and structural motifs in electrode materials—often even in the paramagnetic state [7,8].

For layered lithium–cobalt–nickel–manganese oxides and related cathodes, ultrafast MAS has proven particularly effective. For example, Stoyanova and co-workers used high-speed ^7^Li MAS NMR (with complementary EPR) to connect lithium local structure with electrochemical behaviour in LiCo_1−2x_Ni_x_Mn_x_O_2_ materials, demonstrating that high MAS rates can yield stable, interpretable spectral profiles and resolved intensity distributions even in strongly paramagnetic compositions [9].

Building on these developments, the present work targets a key hardware gap: bringing genuinely high-speed MAS into a compact permanent-magnet geometry. We demonstrate, 50 kHz MAS in a miniaturized setup operating at 1.4 T within an 18 mm bore, establishing a new benchmark for integrating ultrafast MAS capabilities into portable and environmentally relevant, cryogen-free NMR platforms. The performance of the system is demonstrated on paramagnetic lithium-ion battery electrode materials, where the combination of fast MAS and compact-field operation enables spectra that can be directly compared with the literature reports [9] under ultrafast MAS conditions at low magnetic fields.

## 2. Results

### 2.1. Magnet and Probe Configuration

**Figure 1:**
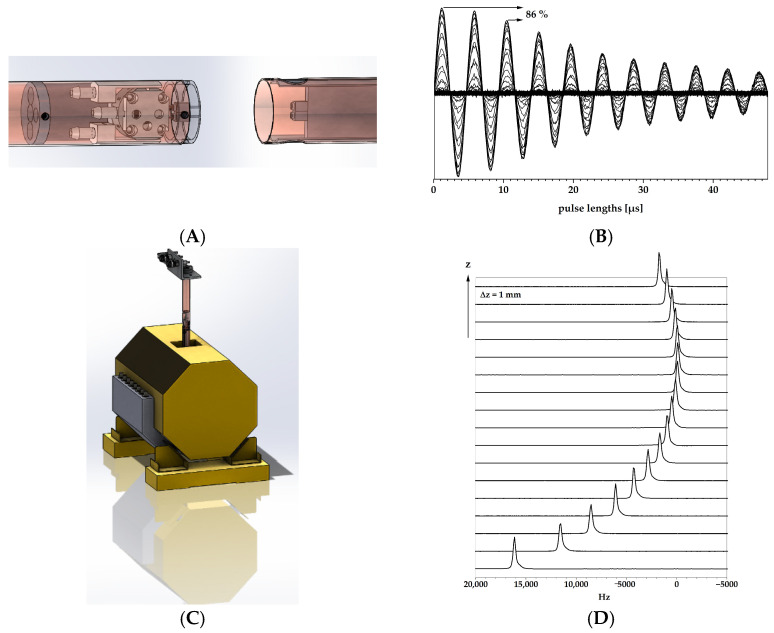
MAS detector with an outer diameter of 18 mm. (**A**) Exploded view showing the probe-head inserted from the top (mechanical turbine including the detection coil) and from bottom (resonant printed-circuit-board section); the two parts are connected inside the 18 mm bore (**C**). (**B**) ^1^H nutation curve showing B_1_ ≈ 0.2 MHz at ~120 W RF power. (**D**) Spectral dependence on probe position (1 mm steps) used to locate the magnet “sweet spot”. The frequency offset Δν (Hz) is given relative to the carrier frequency ν_0_.

The 1.4 T permanent magnet (18 mm bore) and integrated MAS probe-head are shown in Figure 1. Stable field homogeneity was achieved over a <5 mm spherical volume. The probe-head could be repositioned in ±1 mm steps without significant spectral distortion to locate the magnet “sweet spot,” and fine azimuthal rotation enabled precise alignment to the 54.74° magic angle.

### 2.2. Spectroscopic Performance

High-resolution MAS spectra were achieved at 50 kHz spinning speed. For Adamantane, a ^1^H line width of ~8 ppm was achieved. Off-angle spectra demonstrated broadened and asymmetric lines, confirming the criticality of precise angle alignment.

**Figure 2:**
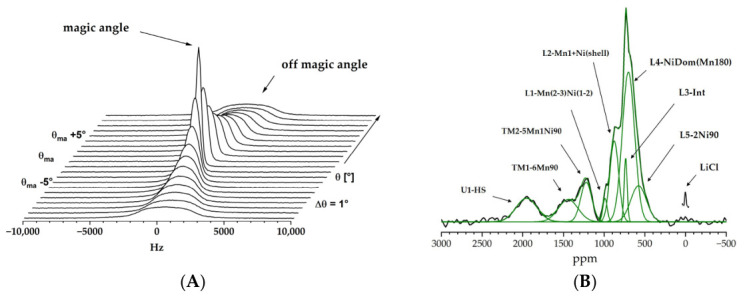
(**A**) Spectral response to magic-angle (MA) adjustment, shown as frequency offset Δν (Hz) relative to the carrier frequency ν_0_ (same definition as in Figure 1D). (**B**) ^7^Li MAS NMR spectrum of the paramagnetic LIB cathode material LiNi_0.5_Mn_0.5_O_2_ acquired on the 1.4 T (18 mm bore) benchtop setup. The improved spectral resolution under high-speed MAS is consistent with ultrafast-MAS low-field spectra reported by Stoyanova et al. [9], see Table 1. A weak additional contribution is observed near ~2000 ppm, which may correspond to low-intensity Li environments and is under ongoing analysis. A LiCl reference trace (external standard) is included for comparison.

As a reference for instrumental resolution, we additionally measured ^7^Li MAS NMR of LiCl under the same probe conditions. The line shows a FWHM linewidth of 15 ppm at 50 kHz MAS (Figure 2B), confirming that the broad features observed for LiNi_0.5_Mn_0.5_O_2_ are dominated by paramagnetic interactions rather than instrument-limited field inhomogeneity.

**Table 1:** Deconvoluted ^7^Li spectrum components of LiNi_0.5_Mn_0.5_O_2_ at 1.4 T (this work) and qualitative assignment following [9].

Component Label	δ/ppm	Site Class	Assignment (Concise)	Motif Detail (from Figure 2B Annotations)
L5-2Ni90	577(79)	Li_Li (layer)	Ni-only closest sites	Li with only Ni^2+^ in the two closest 90° Li–O–TM positions
L4-NiDom(Mn180)	702(8)	Li_Li (layer)	Ni-dominant	Dominantly Ni^2+^ neighbours; Mn^4+^ only in 180° positions
L3-Int	736(1)	Li_Li (layer)	Intermediate component	No explicit neighbour motif indicated in Figure 2B
L2-Mn1+Ni(shell)	876(3)	Li_Li (layer)	Mixed first/second shell	1× Mn^4+^ + several Ni^2+^ in first/second shell
L1-Mn(2-3)Ni(1-2)	989(3)	Li_Li (layer)	Mixed Mn/Ni neighbours	Regular Li layer with 2–3× Mn^4+^ (90°) and 1–2× Ni^2+^ neighbours
TM2-5Mn1Ni90	1221(3)	Li_TM (antisite)	Mn-rich antisite	Li in TM layer; 5× Mn^4+^ + 1× Ni^2+^ via 90° Li–O–TM pathways
TM1-6Mn90	1442(11)	Li_TM (antisite)	Mn-only antisite	Li in TM layer; 6× Mn^4+^ via 90° Li–O–Mn pathways
U1-HS	1953(4)	Unassigned	High-shift feature	High-shift feature; site not assigned (under ongoing analysis)

Figure 2B shows the ^7^Li MAS NMR spectrum of LiNi_0.5_Mn_0.5_O_2_. The spectrum exhibits substantial MAS-induced line narrowing and resolves a dominant broad envelope in the several-hundred-ppm regime. This behaviour is consistent with the low-field ultrafast-MAS data reported by Stoyanova et al., where the stabilized high-speed profile for LiNi_0.5_Mn_0.5_O_2_ is dominated by components in the 300–1000 ppm range (reported to account for ~94% of the intensity), with only a minor contribution in the 1350–1500 ppm region (<6%) [9]. In that work, the 1350–1500 ppm components were assigned to Li environments located in transition-metal layers arising from Li/Ni disorder, whereas the 300–1000 ppm region reflects Li in Li layers experiencing different local TM-neighbour configurations and exchange/dipolar contributions. We additionally observe spectral intensity at very high apparent shifts (around ~2000 ppm) that remains to be further investigated, see Table 1.

## 3. Discussion

To our knowledge, this is the first reported implementation of MAS NMR at 50 kHz in a bore size as small as 18 mm using a permanent magnet.

In established MAS hardware systems, larger rotors are typically operated at lower spinning frequencies (e.g., 7 mm up to ~7 kHz and 4 mm up to ~15 kHz), whereas fast/ultrafast MAS is achieved using smaller rotors (e.g., 1.3 mm up to ~67 kHz, 0.8 mm ~110 kHz, 0.5 mm ~170, and 0.4 over 200 kHz [10,11]). Such conventional solid-state MAS probe families are commonly designed around standard-bore (~54 mm) and wide-bore (~89 mm) magnet geometries [4]. In contrast, achieving high MAS rates in the confined geometry of compact magnets remains comparatively underexplored for <45 mm bore sizes [2].

The demonstrated probe-head concept is broadly applicable to commercial benchtop permanent-magnet systems that provide sufficiently compact access to the magnetic-field “sweet spot” and allow stable routing of drive and bearing gas lines for a miniaturized MAS turbine. In practice, adaptation to a specific instrument would primarily require mechanical customization of the outer probe diameter, insertion length, and mounting/positioning interface to match bore and access constraints, together with system-specific implementation of gas supply and exhaust handling to ensure stable high-speed spinning. The NMR electronics (tuning/matching and console interface) follow standard RF practice; the key enabling feature in the present work is the compact mechanical integration of the MAS turbine and detection coil within a small-bore permanent-magnet geometry, with the stator plug-connected to a printed circuit board that hosts the remaining resonant-circuit components.

The results demonstrate that high-speed MAS can be implemented in compact permanent-magnet geometries, enabling cost-effective solid-state MAS NMR without cryogenics. Because magic-angle optimization here relies on field–bore orthogonality and azimuthal rotation, applying the same alignment strategy to conventional superconducting magnets (with *B_0_* along the bore axis) would require a different and compromised mechanical approach.

An improved spectral resolution due to reduced paramagnetic broadening and high-spinning speed has been clearly monitored at such a low-field benchtop setup. A new weak feature is observed near ~2000 ppm. Its enhanced visibility may reflect the altered balance of paramagnetic broadening and relaxation at low *B_0_* under ultrafast MAS. Future work may address interpreting such additional peak patterns and driving further applications.

## 4. Materials and Methods

### 4.1. Magnet Design and Bore Size

A 1.4 T permanent magnet (Qualion Ltd., formerly Foxboro NMR, Haifa, Israel) with an 18 mm cylindrical bore was used. The field stability and homogeneity were improved using passive shielding and internal homogenization. The magnet geometry allowed insertion of a custom-built MAS probe-head while maintaining stable field conditions in the central measurement region. A heater assembly was used for the magnet temperature stabilization.

### 4.2. MAS Probe and Rotor Design

Because the static magnetic field is orthogonal to the magnet bore axis, the MAS angle can be adjusted by rotating the probe around the bore axis, without requiring additional radial space for turning the stator to a specific angle. This geometry simplifies the mechanical layout compared with conventional MAS probes constrained by a fixed 54.74° rotor relative to the bore axis.

Bore and field orthogonality and sweet spot access from both ends render the use of small-bore permanent magnets relatively efficient. The MAS system was designed for 1.8 mm diameter rotors, with specially miniaturized length (5 mm). Rotors were spun using a compressed air turbine with controlled inlet pressure, with common drive and bearing gas channels. The system sustained spinning rates up to 50 kHz with a sufficient temperature stability and minimal wobble.

### 4.3. Sample Preparation

Proof-of-principle powder samples were filled into 1.8 mm rotors under dry conditions. No additional solvents or dopants were used. As a test application material powder, we used mixed oxides of lithium, nickel, and manganese (LiNi_0.5_Mn_0.5_O_2_) [9].

### 4.4. NMR Spectroscopy

Experiments were conducted using a Varian INOVA 600 MHz console adapted to the 1.4 T probe-head, equivalent to a proton Larmor frequency of ~60 MHz. Pulse calibration (~2.5 µs for 90°), standard nutation, and referencing were performed at room temperature on LiCl. Spectra were recorded under MAS and off-angle conditions.

### 4.5. Data Processing and Analysis

Time domain data were acquired with the Varian INOVA console and exported as complex FIDs. Spectral processing (Fourier transformation, phase correction, and baseline correction) was performed using MestreNova (Mestrelab Research) v14.2.0. The processing software is used for standard post-acquisition analysis and does not require hardware integration into the probe-head or magnet system.

## 5. Conclusions

We report a compact solid-state NMR system operating at 50 kHz MAS within an 18 mm bore of a 1.4 T permanent magnet. The system achieves stable and high-quality spectra, confirming its utility for advanced, yet very affordable and sustainable MAS NMR. This design may catalyze the development of portable MAS NMR for field applications and point-of-care diagnostics. Recent progress in permanent magnets up to 2.1 T renders even ^13^C CP experiments feasible.

## Data Availability

Work did not generate any large scale data.
